# Supplementary material for: High-resolution synthesis of high-density breast mammograms: Application to improved fairness in deep learning based mass detection
Source: Front Oncol. 2023 Jan 23;12:1044496. doi: 10.3389/fonc.2022.1044496 (PMC9899892; doi:10.3389/fonc.2022.1044496)
Supplement: Supplementary file 1 [file DataSheet_1.pdf]

Table A. Performance metrics of the detection models trained with OPTIMAM Hologic dataset. The OPTIMAM Hologic test sets comprise 120 mammograms from the four BI-RADS categories. The upper part of the table correspond to the models trained without BI-RADS D mammograms, meanwhile the lower part corresponds to the models trained using BI-RADS D mammograms (39 scans in total). The metrics reported are the sensitivity, precision and F1-score at 0.75 False Positives per Image (FPPI), and the Area under the Curve of the Free-response Receiver Operating Characteristic (AUC FROC). The mean values and 95% Confidence Intervals of all metrics correspond to the average of five different seeds of each training setup.

	**OPTIMAM Hologic Training Set**									
	BI-RADS D mammograms NOT included in the training									
	**Baseline**		**BC-Aug**		**CS-Aug**		**OP-Aug**		**OP-CS-BC-Aug**	
	Mean	±95% CI	Mean	±95% CI	Mean	±95% CI	Mean	±95% CI	Mean	±95% CI
**OPTIMAM Hologic BI-RADS A**										
Sensitivity @ 0.75 FPPI	0.9711	0.0261	0.9689	0.0285	0.9656	0.0297	0.9644	0.0299	0.9639	0.0304
Precision @ 0.75 FPPI	0.7604	0.0560	0.7499	0.0567	0.7709	0.0574	0.7675	0.0574	0.7761	0.0574
F1-Score @ 0.75 FPPI	0.8140	0.0452	0.8071	0.0458	0.8198	0.0476	0.8160	0.0471	0.8223	0.0475
FROC AUC	0.9488	0.0032	0.9448	0.0063	0.9390	0.0038	0.9367	0.0100	0.9433	0.0050
**OPTIMAM Hologic BI-RADS B**										
Sensitivity @ 0.75 FPPI	0.9364	0.0369	0.9419	0.0338	0.9397	0.0364	0.9381	0.0365	0.9394	0.0346
Precision @ 0.75 FPPI	0.7708	0.0564	0.7681	0.0577	0.7676	0.0598	0.7589	0.0576	0.7738	0.0565
F1-Score @ 0.75 FPPI	0.8074	0.0464	0.8023	0.0455	0.8010	0.0480	0.7994	0.0461	0.8045	0.0459
FROC AUC	0.9030	0.0056	0.9050	0.0024	0.9118	0.0129	0.9047	0.0129	0.8994	0.0076
**OPTIMAM Hologic BI-RADS C**										
Sensitivity @ 0.75 FPPI	0.9625	0.0313	0.9692	0.0282	0.9508	0.0357	0.9650	0.0294	0.9583	0.0329
Precision @ 0.75 FPPI	0.7711	0.0577	0.7684	0.0590	0.7634	0.0602	0.7629	0.0582	0.7795	0.0593
F1-Score @ 0.75 FPPI	0.8171	0.0476	0.8167	0.0472	0.8074	0.0497	0.8105	0.0471	0.8203	0.0484
FROC AUC	0.9300	0.0090	0.9320	0.0067	0.9212	0.0104	0.9306	0.0093	0.9223	0.0050
**OPTIMAM Hologic BI-RADS D**										
Sensitivity @ 0.75 FPPI	0.8517	0.0636	0.8592	0.0618	0.8508	0.0636	0.8567	0.0629	**0.8708**	0.0596
Precision @ 0.75 FPPI	0.7146	0.0703	0.7232	0.0690	0.7032	0.0698	0.7000	0.0693	**0.7224**	0.0683
F1-Score @ 0.75 FPPI	0.7488	0.0655	0.7566	0.0637	0.7414	0.0647	0.7426	0.0642	**0.7596**	0.0626
FROC AUC	0.7971	0.0127	0.7962	0.0179	0.7933	0.0214	0.7986	0.0156	**0.8095**	0.0132
	BI-RADS D mammograms included in the training (39 scans)									
**OPTIMAM Hologic BI-RADS A**										
Sensitivity @ 0.75 FPPI	0.9689	0.0279	0.9683	0.0284	0.9667	0.0295	0.9636	0.0299	0.9717	0.0268
Precision @ 0.75 FPPI	0.7559	0.0569	0.7551	0.0570	0.7689	0.0573	0.7625	0.0569	0.7531	0.0568
F1-Score @ 0.75 FPPI	0.8106	0.0460	0.8096	0.0462	0.8175	0.0471	0.8126	0.0468	0.8089	0.0458
FROC AUC	0.9438	0.0072	0.9418	0.0058	0.9390	0.0111	0.9410	0.0067	0.9472	0.0084
**OPTIMAM Hologic BI-RADS B**										
Sensitivity @ 0.75 FPPI	0.9453	0.0328	0.9408	0.0346	0.9505	0.0301	0.9400	0.0353	0.9420	0.0343
Precision @ 0.75 FPPI	0.7697	0.0572	0.7708	0.0562	0.7789	0.0558	0.7731	0.0573	0.7744	0.0567
F1-Score @ 0.75 FPPI	0.8074	0.0453	0.8056	0.0453	0.8123	0.0440	0.8075	0.0458	0.8064	0.0452
FROC AUC	0.9109	0.0054	0.8995	0.0064	0.9121	0.0059	0.9015	0.0128	0.9037	0.0074
**OPTIMAM Hologic BI-RADS C**										
Sensitivity @ 0.75 FPPI	0.9650	0.0312	0.9725	0.0260	0.9633	0.0314	0.9608	0.0308	0.9692	0.0277
Precision @ 0.75 FPPI	0.7644	0.0613	0.7773	0.0579	0.772	0.0606	0.7732	0.058	0.7600	0.0587
F1-Score @ 0.75 FPPI	0.8120	0.0482	0.8232	0.0459	0.8168	0.0483	0.8161	0.0477	0.8108	0.0466
FROC AUC	0.9409	0.0055	0.9494	0.0022	0.9300	0.0071	0.9295	0.0129	0.9339	0.0122
**OPTIMAM Hologic BI-RADS D**										
Sensitivity @ 0.75 FPPI	0.8608	0.0618	**0.8742**	0.0594	0.8609	0.062	**0.8767**	0.0582	0.8692	0.0604
Precision @ 0.75 FPPI	0.7128	0.0689	**0.7247**	0.0677	0.7112	0.0694	**0.7210**	0.0678	0.7176	0.0687
F1-Score @ 0.75 FPPI	0.7515	0.0637	**0.7639**	0.0621	0.7498	0.0642	**0.7596**	0.0619	0.7562	0.0630
FROC AUC	0.8060	0.0140	**0.8110**	0.0070	0.8016	0.0173	**0.8075**	0.0198	0.8076	0.0084

Table B. Performance metrics of the detection models trained with OPTIMAM Hologic dataset. The INbreast test set comprise 116 mammograms from all four BI-RADS categories. The upper part of the table correspond to the models trained without BI-RADS D mammograms, meanwhile the lower part corresponds to the models trained using BI-RADS D mammograms (39 scans in total). The metrics reported are the sensitivity, precision and F1-score at 0.75 False Positives per Image (FPPI), and the Area under the Curve of the Free-response Receiver Operating Characteristic (AUC FROC). The mean values and 95% Confidence Intervals of all metrics correspond to the average of five different seeds of each training setup.

	**OPTIMAM Hologic Training Set**									
	BI-RADS D mammograms NOT included in the training									
	**Baseline**		**BC-Aug**		**CS-Aug**		**OP-Aug**		**OP-CS-BC-Aug**	
	Mean	±95% CI	Mean	±95% CI	Mean	±95% CI	Mean	±95% CI	Mean	±95% CI
**Inbreast Dataset**										
Sensitivity @ 0.75 FPPI	0.8536	0.0655	**0.9034**	0.0528	0.8530	0.0653	0.8704	0.0612	0.8953	0.0551
Precision @ 0.75 FPPI	0.7251	0.0742	**0.7513**	0.0681	0.7174	0.0734	0.7310	0.0711	0.7599	0.0690
F1-Score @ 0.75 FPPI	0.7546	0.0687	**0.7864**	0.0605	0.7504	0.0677	0.7638	0.0649	0.7905	0.0619
FROC AUC	0.8151	0.0258	**0.8566**	0.0375	0.8112	0.0401	0.8345	0.0342	0.8447	0.0215
	BI-RADS D mammograms included in the training (39 scans)									
**Inbreast Dataset**										
Sensitivity @ 0.75 FPPI	0.8975	0.0552	0.9025	0.0550	0.8352	0.0669	**0.8972**	0.0548	0.8872	0.058
Precision @ 0.75 FPPI	0.7462	0.0687	0.7493	0.0682	0.7029	0.0748	**0.7513**	0.0687	0.7458	0.0703
F1-Score @ 0.75 FPPI	0.7821	0.0614	0.7875	0.0610	0.7331	0.0692	**0.7855**	0.0616	0.7782	0.0634
FROC AUC	0.8471	0.0132	0.8488	0.0202	0.7902	0.0592	**0.8616**	0.0279	0.8429	0.0207

Table C. Performance metrics of the detection models trained with INbreast dataset. The OPTIMAM Hologic test sets comprise 120 mammograms from the four BI-RADS categories. The upper part of the table correspond to the models trained without BI-RADS D mammograms, meanwhile the lower part corresponds to the models trained using all the BI-RADS D mammograms available in INbreast (8 scans in total). The metrics reported are the sensitivity, precision and F1-score at 0.75 False Positives per Image (FPPI), and the Area under the Curve of the Free-response Receiver Operating Characteristic (AUC FROC). The mean values and 95% Confidence Intervals of all metrics correspond to the average of five different seeds of each training setup.

	**INbreast Training Set**									
	BI-RADS D mammograms NOT included in the training									
	**Baseline**		**BC-Aug**		**CS-Aug**		**OP-Aug**		**OP-CS-BC-Aug**	
	Mean	±95% CI	Mean	±95% CI	Mean	±95% CI	Mean	±95% CI	Mean	±95% CI
**OPTIMAM Hologic BI-RADS A**										
Sensitivity @ 0.75 FPPI	0.7411	0.0784	0.6661	0.0845	0.7539	0.0772	0.7650	0.0756	0.7144	0.0805
Precision @ 0.75 FPPI	0.5467	0.0720	0.5091	0.0752	0.5711	0.0720	0.5839	0.0716	0.5533	0.0741
F1-Score @ 0.75 FPPI	0.6021	0.0704	0.5572	0.0755	0.6262	0.0704	0.6383	0.0696	0.6009	0.0731
FROC AUC	0.6473	0.0165	0.5652	0.0357	0.6543	0.0227	0.6678	0.0193	0.6292	0.0350
**OPTIMAM Hologic BI-RADS B**										
Sensitivity @ 0.75 FPPI	0.6897	0.0786	0.5989	0.0842	0.6803	0.0797	0.6950	0.0774	0.6633	0.0800
Precision @ 0.75 FPPI	0.5693	0.0745	0.5197	0.0794	0.5686	0.0762	0.5815	0.0742	0.5640	0.0762
F1-Score @ 0.75 FPPI	0.5924	0.0708	0.5338	0.0770	0.5872	0.0723	0.6034	0.0705	0.5801	0.0725
FROC AUC	0.6056	0.0338	0.5266	0.0226	0.6051	0.0217	0.6122	0.0304	0.5876	0.0358
**OPTIMAM Hologic BI-RADS C**										
Sensitivity @ 0.75 FPPI	0.7250	0.0794	0.6816	0.0816	0.7192	0.0796	0.7483	0.0776	0.7367	0.0780
Precision @ 0.75 FPPI	0.5372	0.0724	0.5274	0.0747	0.5551	0.0742	0.5893	0.0746	0.6061	0.0754
F1-Score @ 0.75 FPPI	0.5889	0.0704	0.5701	0.0733	0.6027	0.0727	0.6322	0.0719	0.6396	0.0727
FROC AUC	0.5971	0.0176	0.5649	0.0652	0.6005	0.0439	0.6411	0.0045	0.6512	0.0167
**OPTIMAM Hologic BI-RADS D**										
Sensitivity @ 0.75 FPPI	0.5133	0.0901	0.4133	0.0878	0.4892	0.0889	**0.5367**	0.0892	0.5058	0.0891
Precision @ 0.75 FPPI	0.4087	0.0794	0.3439	0.0783	0.3894	0.0776	**0.4568**	0.0824	0.4337	0.0820
F1-Score @ 0.75 FPPI	0.4388	0.0804	0.3629	0.0792	0.4169	0.0784	**0.4779**	0.0822	0.4520	0.0819
FROC AUC	0.4259	0.0286	0.3493	0.0456	0.4009	0.0476	**0.4541**	0.0271	0.4365	0.0301
	BI-RADS D mammograms included in the training (8 scans)									
**OPTIMAM Hologic BI-RADS A**										
Sensitivity @ 0.75 FPPI	0.7595	0.0763	0.6892	0.0829	0.7458	0.0779	0.7800	0.0739	0.6847	0.0831
Precision @ 0.75 FPPI	0.5722	0.0718	0.5175	0.074	0.5683	0.0732	0.5924	0.0704	0.5175	0.0744
F1-Score @ 0.75 FPPI	0.6264	0.0699	0.5700	0.0739	0.6200	0.0715	0.6476	0.0681	0.5672	0.0741
FROC AUC	0.6731	0.0217	0.5931	0.0375	0.6582	0.0172	0.6735	0.0185	0.5782	0.0345
**OPTIMAM Hologic BI-RADS B**										
Sensitivity @ 0.75 FPPI	0.7125	0.0770	0.6250	0.0831	0.6911	0.0784	0.7006	0.0765	0.6183	0.0831
Precision @ 0.75 FPPI	0.5799	0.0729	0.5267	0.0781	0.5626	0.0739	0.5861	0.0739	0.5168	0.0773
F1-Score @ 0.75 FPPI	0.6078	0.0692	0.5445	0.0753	0.5887	0.0704	0.6045	0.0693	0.5360	0.0745
FROC AUC	0.6356	0.0168	0.5476	0.0264	0.6184	0.0410	0.6146	0.0361	0.5296	0.0295
**OPTIMAM Hologic BI-RADS C**										
Sensitivity @ 0.75 FPPI	0.7575	0.0759	0.7300	0.0784	0.7217	0.0800	0.7967	0.0710	0.7258	0.0784
Precision @ 0.75 FPPI	0.5762	0.0717	0.5695	0.0738	0.5477	0.0739	0.6247	0.0706	0.5705	0.0742
F1-Score @ 0.75 FPPI	0.6260	0.0689	0.6122	0.0712	0.5977	0.0724	0.6709	0.0666	0.6113	0.0716
FROC AUC	0.6428	0.0157	0.6209	0.0301	0.6171	0.0442	0.6857	0.0190	0.6082	0.0444
**OPTIMAM Hologic BI-RADS D**										
Sensitivity @ 0.75 FPPI	0.5517	0.0892	0.4608	0.0892	0.4850	0.0893	**0.5875**	0.0880	0.4558	0.0890
Precision @ 0.75 FPPI	0.4521	0.0804	0.3831	0.0801	0.3811	0.0776	**0.4894**	0.0808	0.3797	0.0802
F1-Score @ 0.75 FPPI	0.4803	0.0807	0.4038	0.0807	0.4113	0.0789	**0.5164**	0.0805	0.4002	0.0808
FROC AUC	0.4459	0.0172	0.3964	0.0324	0.4067	0.0460	**0.4884**	0.0263	0.3974	0.0402

Table D. Statistical significance tests of the detection models trained with OPTIMAM Hologic dataset. The Area under the Curve of the Free-response Receiver Operating Characteristic (AUC FROC) is compared to the DeLong's AUC obtained with a maximum of 10 False Postives per Image (FPPI). The *p-values* are obtained using DeLong methodology with a maximum of 10 FFPI. A detection model with a p-value < 0.05 is statistically different from its corresponding baseline. The OPTIMAM Hologic test set comprises 120 mammograms from high density mammograms from BI-RADS D category. The INbreast test set comprise 116 mammograms from all four BI-RADS categories.

**OPTIMAM Hologic**	**Baseline**		**BC-Aug**		**CS-Aug**		**OP-Aug**		**OP-CS-BC-Aug**	
Real BI-RADS D in training	No	Yes	No	Yes	No	Yes	No	Yes	No	Yes
OPTIMAM Hologic BI-RADS D										
FROC AUC	79.71%	80.60%	79.62%	**81.10%**	79.33%	80.16%	79.86%	80.75%	**80.95%**	80.76%
	(78.44, 80.98)	(79.20, 82.00)	(77.83, 81.41)	**(80.40, 81.80)**	(77.19, 81.47)	(78.43, 81.89)	(78.30, 81.42)	(78.77, 82.73)	**(79.63, 82.27)**	(79.92, 81.60)
DeLong's AUC (max 10 FPPI)	80.02%	80.71%	79.23%	**81.13%**	79.17%	80.08%	80.07%	80.57%	**80.68%**	80.59%
	(77.25, 82.78)	(77.98, 83.44)	(76.46, 82.00)	**(78.47, 83.80)**	(76.34, 81.99)	(77.33, 82.83)	(77.34, 82.81)	(77.88, 83.26)	**(77.99, 83.37)**	(77.88, 83.30)
p-value	Reference	Reference	0.0064	**0.2277**	1.63e-06	8.96e-06	0.8269	0.5599	**0.0696**	0.7921
INbreast dataset										
FROC AUC	81.51%	84.71%	**85.66%**	84.88%	81.12%	79.02%	83.45%	**86.16%**	84.47%	84.29%
	(78.93, 84.09)	(83.39, 86.03)	**(81.91, 89.41)**	(82.86, 86.90)	(77.11, 85.13)	(73.10, 84.94)	(80.03, 86.87)	**(83.37, 88.95)**	(82.32, 86.62)	(82.22, 86.36)
DeLong's AUC (max 10 FPPI)	80.47%	83.68%	**83.30%**	82.92%	80.17%	77.11%	81.90%	**84.62%**	82.45%	82.57%
	(77.56, 83.38)	(81.08, 86.28)	**(80.67, 85.92)**	(80.24, 85.59)	(77.25, 83.08)	(73.98, 80.25)	(79.09, 84.70)	**(82.01, 87.23)**	(79.73, 85.18)	(79.85, 85.30)
p-value	Reference	Reference	**0.0002**	0.1666	0.4937	6.18e-10	6.08e-05	**0.0041**	0.0008	0.0162

Table E. Statistical significance tests of the detection models trained with INbreast dataset. The Area under the Curve of the Free-response Receiver Operating Characteristic (AUC FROC) is compared to the DeLong's AUC obtained with a maximum of 10 False Postives per Image (FPPI). The *p-values* are obtained using DeLong methodology with a maximum of 10 FFPI. A detection model with a p-value < 0.05 is statistically different from its corresponding baseline. The OPTIMAM Hologic test set comprises 120 mammograms from high density mammograms from BI-RADS D category.

**INBreast**	**Baseline**		**BC-Aug**		**CS-Aug**		**OP-Aug**		**OP-CS-BC-Aug**	
Real BI-RADS D in training	No	Yes	No	Yes	No	Yes	No	Yes	No	Yes
OPTIMAM Hologic BI-RADS D										
FROC AUC	42.59%	44.59%	34.93%	39.64%	40.09%	40.67%	**45.41%**	**48.84%**	43.65%	39.74%
	(39.73, 45.45)	(42.87, 46.31)	(30.37, 39.49)	(36.40, 42.88)	(35.33, 44.85)	(36.07, 45.27)	**(42.70, 48.12)**	**(46.21, 51.47)**	(40.64, 46.66)	(35.72, 43.76)
DeLong's AUC (max 10 FPPI)	41.33%	45.30%	35.77%	39.84%	41.37%	40.48%	**45.06%**	**49.01%**	43.69%	39.53%
	(37.88, 44.77)	(41.87, 48.73)	(32.37, 39.18)	(36.34, 43.34)	(37.91, 44.84)	(37.05, 43.91)	**(41.54, 48.57)**	**(45.52, 52.49)**	(40.17, 47.22)	(36.00, 43.06)
p-value	Reference	Reference	2.13e-13	2.29e-11	0.9370	3.40e-16	**4.58e-22**	**1.43e-27**	2.32e-11	9.40e-10
